# Controlling the Colonization of *Clostridium perfringens* in Broiler Chickens by an Electron-Beam-Killed Vaccine

**DOI:** 10.3390/ani11030671

**Published:** 2021-03-03

**Authors:** Palmy R. Jesudhasan, Sohini S. Bhatia, Kirthiram K. Sivakumar, Chandni Praveen, Kenneth J. Genovese, Haiqi L. He, Robert Droleskey, Jack L. McReynolds, James A. Byrd, Christina L. Swaggerty, Michael H. Kogut, David J. Nisbet, Suresh D. Pillai

**Affiliations:** 1 Poultry Production and Product Safety, USDA-ARS, 1260 W Maple St., O-306 POSC Building, University of Arkansas, Fayetteville, AR 72701, USA; Palmy.Jesudhasan@usda.gov; 2 National Center for Electron Beam Research, An IAEA Collaborating Centre for Electron Beam Technology, Texas A&M University, College Station, TX 77843, USA; sohinibhatia@tamu.edu (S.S.B.); kirthiramsivakumar@gmail.com (K.K.S.); Chandni.vs@gmail.com (C.P.); 3 Food and Feed Safety Research Unit, USDA-ARS, 2881 F and B Rd, College Station, TX 77845, USA; kenneth.genovese@usda.gov (K.J.G.); haiqi.he@usda.gov (H.L.H.); bob.droleskey@usda.gov (R.D.); Allen.Byrd2@usda.gov (J.A.B.); christi.swaggerty@usda.gov (C.L.S.); mike.kogut@usda.gov (M.H.K.); david.nisbet@usda.gov (D.J.N.); 4 Arm & Hammer Animal and Food Production, Church & Dwight Co. Inc., 6935 Vista Drive, West Des Moines, IA 50266, USA; Jackson.McReynolds@churchdwight.com

**Keywords:** electron beam irradiation, eBeam, eBeam-killed vaccine, *Clostridium perfringens*, broiler chickens, in ovo vaccination, embryo vaccination, challenge

## Abstract

**Simple Summary:**

*Clostridium perfringens* (Cp) is a bacterium that causes necrotic enteritis in chickens and is responsible for an economic loss of about 6 billion U.S. dollars in the poultry industry worldwide. Consumption of Cp contaminated chicken meat causes foodborne illnesses in humans. Although Cp can be controlled in chickens by administering antibiotics through feed and water, the ban on the antibiotics owing to concerns on antibiotic resistance has created the need to identify alternative control approaches. As vaccination could be used as an alternative, we used electron beam irradiation (eBeam) to kill the bacterium and use the killed cells as vaccine to control the colonization of Cp in broiler chickens. In this study, we exposed three different strains of Cp to eBeam irradiation and used them as a vaccine to day-18 embryos. After the embryos hatched, the birds in each treatment were segregated into two groups for live Cp challenge at two time points. The results indicate that the vaccine effectively controlled the colonization of all three strains of Cp when challenged with live Cp, indicating that the vaccinated birds had acquired immunity. The current approach can reduce Cp colonization in chickens, thereby reducing economic loss.

**Abstract:**

*Clostridium perfringens* (Cp) is a Gram-positive anaerobe that is one of the causative agents of necrotic enteritis (NE) in chickens, which leads to high mortality. Owing to the ban of administering antibiotics in feed to chickens, there has been an increase in the number of NE outbreaks all over the world, and the estimated loss is approximately 6 billion U.S. dollars. The best alternative method to control NE without antibiotics could be vaccination. In this study, we exposed three different strains of Cp to electron beam (eBeam) irradiation to inactivate them and then used them as a killed vaccine to control the colonization of Cp in broiler chickens. The vaccine was delivered to 18-day old embryos in ovo and the chickens were challenged with the respective vaccine strain at two different time points (early and late) to test the protective efficacy of the vaccine. The results indicate that an effective eBeam dose of 10 kGy inactivated all three strains of Cp, did not affect the cell membrane or epitopes, induced significant levels of IgY in the vaccinated birds, and further reduced the colonization of Cp strains significantly (*p* < 0.0001) in late challenge (JGS4064: 4 out of 10; JGS1473: 0 out of 10; JGS4104: 3 out of 10). Further studies are necessary to enhance the efficacy of the vaccine and to understand the mechanism of vaccine protection.

## 1. Introduction 

*Clostridium perfringens* (Cp) is a Gram-positive, rod-shaped, spore-forming, anaerobic bacterium that causes several human and animal diseases and is considered the most important clostridial pathogen of poultry [1]. Cp is also a foodborne pathogen causing 19 outbreaks and 478 illnesses in the United States annually (CDC 2019). This bacterium, along with other predisposing factors, causes necrotic enteritis (NE) in broiler chickens aged 2 to 6 weeks old [2,3] and is associated with mucosal damage in the intestine [4]. NE causes up to 30% mortality in broiler chickens, costing the worldwide poultry industry approximately $6 billion U.S. dollars annually [5], and causes an estimated $342 million loss in the United States due to foodborne illnesses [6]. For more than forty years, poultry feed and water have been supplemented with antibiotics to improve poultry growth performance and feed efficiency, with the added benefit of protecting poultry from the harmful effects of enteric microorganisms [7]. However, concerns about increased spread of antibiotic resistance markers [8,9] have led to the discontinued use of antibiotic growth promoters in much of the global poultry industry. With the decreased use of antimicrobial drugs, however, there has been an increase of NE in poultry from approximately 4% to 12% [10,11,12].

Cp produces several potent protein toxins that are important to its virulence and, based on these toxins, Cp strains are classified into seven types, designated A–G [13,14]. Types A and C are known to cause NE in chickens [15]. The α-toxin, a major secretory protein of Cp produced by all Cp strains, was once considered the essential virulence factor in the pathogenesis of NE [4]; however, numerous studies have demonstrated that α-toxin knockout mutants are still capable of inducing disease [3,12,16,17]. Other studies have reported that NetB toxin, and not α-toxin, is the essential virulence factor in NE infection [15,16]. Cp strains producing both NetB and TpeL are considered more virulent in producing NE [18,19]. There is conflicting evidence of these toxins’ prevalence and importance in causing NE [17,20,21]. It is very likely that multiple virulence factors function synergistically to induce disease.

Recombinant Cp α-toxin and NetB vaccines have been found to give partial protection against NE [1,22,23,24,25,26]. Owing to the differential presence and absence of toxin genes among the individual Cp strains used, a solution to the NE problem is still elusive [1,14]. Immunization of chickens against the disease is considered an alternative strategy to control NE in poultry. Killed whole cell bacterial vaccines are one of the best methods to control bacterial infections [27], but, currently, no commercial vaccines are available to control NE.

We have previously developed an efficacious, eBeam-killed, whole cell *Salmonella* Enteritidis vaccine [28] to protect against *S*. Enteritidis colonization and shedding in poultry. The vaccine significantly enhanced immunity in vaccinated laying hens by reducing colonization and shedding of *S.* Enteritidis when challenged with *S*. Enteritidis [29]. The eBeam-killed bacterial cells cannot multiply, they remain structurally intact, and can elicit an immune response [29,30,31]. eBeam technology, which can deliver highly precise eBeam doses, is in use worldwide commercially for the sterilization of single-use medical devices, food pasteurization, and other applications. This same technology can be re-purposed to inactivate large volumes of bacterial preparations to develop killed vaccines that would be effective against Cp.

The objective of this study was to develop an eBeam-killed vaccine to control the colonization of Cp and NE in broiler chickens. Our hypothesis was that, if a cocktail of strains were used for a vaccine formulation, it could protect chickens from NE caused by Cp colonization, as the cocktail would avoid the stain-to-strain variations in protection seen in other studies. Before developing a vaccine based on a cocktail of strains, it was important to understand the efficacy of the strains individually. Therefore, in this study, we used three different Cp strains to understand the efficacy of individual eBeam-killed-Cp preparations. The rationale of the study was that immunizing broiler chicken embryos in ovo with eBeam-killed-CP vaccine would offer enhanced immunity against Cp and protect chickens from CP colonization from the day of hatch. The eBeam-killed vaccine approach could overcome concerns about introducing live attenuated pathogenic strains (as vaccines) into food animals, as well as eliminate the need for the use of low-dose antimicrobials in food animal production systems.

## 2. Materials and Methods

### 2.1. eBeam Dose Optimization

Bacterial isolates: The NE poultry isolates (JGS4064—Group A and *cpb*2 negative, *netB* negative; JGS4104 Group A and *cpb*2 positive, *netB* negative) and normal flora (JGS1473—(Group A and *cpb*2 positive, *netB* negative)) of Cp strains used in this study were obtained from Glenn Songer (University of Arizona). All three strains have been characterized [32,33]. Individual cultures of each Cp strain were incubated anaerobically overnight in fluid thioglycolate broth (FTG) at 37 °C in an anaerobic chamber (5% hydrogen/5% carbon dioxide/90% nitrogen).

Determination of D_10_ value: Overnight cultures of Cp strains were aliquoted (3 mL) into sterile Whirl-Pak^®^ polyethylene bags (Nasco, Fort Atkinson, WI, USA). Each bag was flattened to enable uniform eBeam dosing and then heat-sealed. Sets of three bags (replicates from an overnight culture) were exposed to multiple eBeam doses. One set was left untreated as a control. After eBeam exposure, ten-fold serial dilutions were conducted in phosphate-buffered saline PBS pH 7.4, plated onto blood agar plates, and incubated anaerobically at 37 °C for 24 h, after which colonies were enumerated and the number of surviving colony forming units (CFU) was calculated. The D_10_ value (dose required to reduce a bacterial culture by one log) for each Cp strain was calculated as the inverse of the slope of the regression line obtained from the inactivation curve. Regression analysis was performed in Microsoft Excel.

Vaccine preparation: All three Cp strains were grown individually, anaerobically in FTG broth overnight at 37 °C. Individually, 10 mL of approximately 10^8^ CFU/mL cultures was placed in plastic bags and heat-sealed, with care taken to ensure there were no air bubbles or leakage of the bags. Sample bags were then double sealed in a new plastic bag and placed inside a 95 kPa biohazard transport bags. Samples were transported on blue ice from the laboratory to the National Center for Electron Beam Research on the Texas A&M University campus. Total time of transport was less than 30 min.

Electron beam treatment: Samples were subjected to eBeam treatment. Briefly, eBeam dosing was carried out using a high-energy (10 MeV), 18 kW linear accelerator (LINAC). The delivered dose was measured using four alanine dosimeters (BioMax, Bruker BioSpin MRI, Inc., Billerica, MA, USA) placed on the top and bottom of the packaged samples. The absorbed dose with the dosimeters was measured using an electron paramagnetic resonance (EPR) E-scan analyzer (Bruker BioSpin MRI Inc., Billerica, MA). The reported absorbed dose was the average of the four dosimeters. An extensive set of preliminary studies were performed to ensure that the sample bag configuration yielded a dose-uniformity ratio (DUR) as close to 1.0 as possible. The DUR (maximum dose divided by the minimum dose received) is an important criterion when performing irradiation experiments to ensure the dose uniformity within the sample. The cells were exposed to a target dose of 10 kGy (kilo Grays) based on the determined D_10_ value for each strain. The delivered dose for each experiment was measured with the use of the Bruker E-scan analyzer. Aliquots of the irradiated Cp cells were stored at 4 °C until utilized for different assays to confirm inactivation, verification of membrane integrity, electron microscopic analysis, and vaccination purposes.

### 2.2. Characterization of E-Beam-Killed C. perfringens Cells

Studies were performed to verify that the eBeam-killed bacterial cells would not replicate either in vitro or in vivo. For the in vitro verification, aliquots of the irradiated cells were inoculated in FTG and blood agar plates incubated anaerobically at 37 °C and room temperature for up to 2 weeks. For the in vivo verification, 18-day-old chicken specific-pathogen free (SPF) embryos were obtained commercially from a local commercial facility. The large end of all eggs was wiped with 70% ethanol and gently scored with an 18-gauge needle. Then, 100 µL of eBeam-killed cells (1 × 10^6^ CFU) or sham (FTG) was administered in ovo into the amnion using a 1 mL tuberculin syringe and a 25-gauge needle equipped with a modified needle guard to limit all injections to a depth of 3 cm. Following treatment injection to individual embryos, injection sites on all eggs were covered with melted paraffin using a cotton swab and transferred to a hatcher maintained at 38 ± 0.5 °C and 55–75% humidity. On day-of-hatch, five chicks/treatments were placed in individual rearing pens at appropriate rearing temperatures on clean pine shaving litter material. Birds were reared in 2.4 m × 1.2 m pens, allowing 0.12 m^2^ pen space per bird, and the chicks were maintained for 2 weeks. Diet provided was according to National Research Council’s guidelines for broiler chicks with water and 55% wheat/corn-based broiler starter diet fed ad libitum. A high wheat diet was used as high concentrations of wheat in the diet have been reported to aggravate outbreaks of NE [34]. The birds were necropsied and examined for Cp colonization in the small intestine by dissecting a 6-inch section of small intestine cranial to Meckel’s diverticulum, placed in 10 mL of anaerobic FTG, stomached for 30 s, and 0.5 mL of gut contents was removed and placed into 4.5 mL of FTG. Three 10-fold serial dilutions were performed and plated onto blood agar plates to confirm the absence of any Cp colonies.

Bacterial cell membrane integrity assay: A mixture of reagent A and reagent B (1:1 ratio) 2× staining solution from LIVE/Dead^®^BacLight™ bacterial viability kit (ThermoFisher, Waltham, MA, USA) was prepared according to the manufacturer’s instructions. Briefly, one hundred microliters of 2× staining solution was mixed with either 100 µL eBeam-killed or non-irradiated Cp cells in a well of a 96-well plate and incubated at room temperature for 15 min. The fluorescence intensity at a wavelength centered at about 530 nm (emission 1; green) and 630 nm (emission 2; red) for each well of the entire plate was measured using a fluorescent spectrophotometer. Five replicate wells were used for each strain. Differences in the fluorescence intensity at 530 nm between treatments indicated the integrity of the cell membrane.

AlamarBlue assay: The alamarBlue^TM^ cell viability assay was performed according to the manufacturer’s instructions (ThermoFisher). This assay was used to measure the metabolic activity with eBeam-treated Cp and non-irradiated Cp. Briefly, resazurin is reduced to resorufin in the presence of a reducing environment, such as the case in a metabolically active bacterium. For this experiment only, Cp strains were grown anaerobically in reinforced clostridial medium (RCM) rather than FTG. This was done because FTG contains ingredients such as sodium thioglycolate and resazurin that interfere with assay performance. The metabolic activity was measured after eBeam treatment. Then, 90 µL of each irradiated sample was added to a 96-well plate along with 10 µL of 10× alamarBlue™ reagent. Non-irradiated Cp was used as control. The plate was incubated in the dark at 37 °C for 1 h and fluorescence was read using a plate reader at an excitation wavelength between 540 and 570 and emission wavelength between 580 and 610 nm.

Sample preparation for scanning and transmission electron microscope analyses: Aliquots of the eBeam-irradiated and non-irradiated Cp cells were preserved for ultrastructural examination by mixing equal volumes of sample with a fixative containing 4% glutaraldehyde prepared in 100 mM phosphate and 100 mM sucrose, pH 7.4. After initial fixation, samples were rinsed twice in a wash buffer (50 mM phosphate, 50 mM sucrose, pH 7.4) by centrifugation at 1600× *g* for 10 min and resuspended in 1 mL of buffer. For those cells examined using SEM, a 50 µL aliquot of the sample was placed on 0.1% poly-L-lysine-coated glass coverslips and incubated at room temperature for 60 min. The sample was fixed to the coverslips by immersion in 3% glutaraldehyde fixative for 60 min, followed by postfixation in 1% OsO_4_ prepared in 100 mM phosphate and 100 mM sucrose, pH 7.4, for 90 min at 4 °C. The coverslips were dehydrated and critical point dried in CO_2_. The dried samples were then sputter-coated with gold and examined in a Hitachi H7110 Scanning-Transmission Electron Microscope (STEM) (Hitachi High-Tech America, Schaumburg, IL, USA). The remaining portions of the samples were pelleted in 2.5% agar, postfixed in 1% OsO_4_ as above, and poststained overnight in 0.5% aqueous uranyl acetate, dehydrated and embedded in Mollenhauer’s mixture of epoxy resin [35]. Thin sections (75 nm) of embedded samples were examined in the same electron microscope as cited above operating in transmission mode.

### 2.3. Embryo Vaccination

Bird studies were performed at the USDA-Southern Plains Area Research Center in College Station under the USDA/SPARC Institutional Animal Care and Use Committee approved procedure. Cobb × Ross straight run broiler embryos were obtained commercially from a local commercial facility on day 18 of embryogenesis. Eggs were candled for viability prior to placement in hatching cabinets in all experiments.

A total of 330 eggs were used for this study; Table 1 (six groups: negative control, positive control, challenge-eyes control, eBeam JGS4064, eBeam JGS1473, and eBeam JGS4104). Each treatment group contained 55 embryos (5 extra embryos were included to account for variation in hatchability), but after hatch, only 50 birds were maintained (25 birds for early-challenge and 25 birds for late-challenge). Embryo vaccination was explained earlier under Section 2.2. Characterization of E-beam-killed *C. perfringens* cells. Out of 25 birds, blood was collected from 15 birds and 10 birds were used for bacterial colonization analysis. All 25 birds were used for lesion scoring and weight sampling. Individual groups were placed in separate hatching trays after vaccination and placed to hatch in the same hatching cabinet to provide identical hatching conditions. On day-of-hatch, the chicks hatched in each treatment group were further divided equally into two groups (early and late challenge) and placed in individual rearing pens on clean pine shaving litter material at appropriate rearing temperatures. Positive and negative control birds were kept in individual rooms separate from the other groups. A commercial bursal disease vaccine (Bursa-Vac, Merck Animal Health, Summit, NJ, USA) was administered to birds at 10× the recommended dose via the ocular route at day 18 for early-challenge and day 25 for late-challenge to immunocompromise the birds and aid in the development of NE [36]. Birds were challenged with the individual strains once daily by oral gavage (3 mL per bird) with a stock culture of 1 × 10^7^ CFU of Cp/mL for 3 consecutive days, beginning on day 18 for early-challenge and day 25 for late-challenge. Positive control birds were challenged with a mixture of all three strains of Cp (1 × 10^7^ CFU^)^. The birds were necropsied on day 22 for early-challenge and day 29 for late-challenge by cervical dislocation and examined for Cp colonization in the ileum by dissecting a 6-inch section of ileum, placed in 10 mL of anaerobic FTG, stomached for 30 s, and 0.5 mL of gut contents were removed and placed into 4.5 mL of FTG. Three 10-fold serial dilutions were performed and plated onto Shahidi–Ferguson-perfringens (SFP) agar to count the vegetative cells of Cp colonies and the plates were incubated in an anaerobic chamber at 37 °C. Blood sample (1 to 2 mL) was drawn from the vein of chickens and transferred to Vacutainer^®^ plastic tubes to collect serum.

### 2.4. Measurement of IgY Antibody Titers

The titer of total serum IgY antibodies was measured with the use of direct enzyme-linked immunosorbent assay (ELISA). Briefly, wells of ninety-six-well polystyrene microtiter plates were coated with 100 µL of respective Cp strain, diluted in coating buffer (50 mM carbonic acid buffer, pH 9.6), and incubated at 4 °C overnight. The plates were blocked with 200 µL of PBS-T containing 5% skim milk at 4 °C overnight. Sera from 15 birds were pooled from each treatment group and diluted 1:10,000 with coating buffer, and 100 µL of each pool was added in triplicate to a 96-well and incubated for 3 h at room temperature, and then washed three times with PBS-T. One hundred microliters of rabbit anti-chicken IgY-HRP antibody (Invitrogen) diluted 1:4000 with blocking buffer was added to each well and incubated for 1 h at 37 °C. After incubation at 37 °C for 1 h, the plates were washed three times with PBS-T, followed by the addition of 100 µL of *o*-phenylenediamine dihydrochloride (OPD)/well (1 mg/mL: made with SIGMAFAST™ OPD two-tablet sets) for the detection of bound IgY. The optical density (OD) at 450 nm of each well was measured using the Wallac Victor 1420 Multilabel counter (Perkin Elmer, Boston, MA, USA). The OD values were recorded as the mean of triplicate wells.

Pre-challenge sera samples were obtained on day 18 for early-challenge and day 25 for late-challenge chicks that had been immunized with specific strains of eBeam-killed Cp vaccine on 18-day-old embryogenesis and non-immunized birds (negative and positive control). The post-challenge sera samples were obtained on day 22 for early-challenge and day 29 for late-challenge from immunized and non-immunized positive and negative control (no challenge) chicks. Chicks in all groups except negative control were challenged with a respective strain of Cp for three consecutive days (18, 19, and 20 for early-challenge, and 25, 26, and 27 for late-challenge), but the positive control was challenged with a mixture of all three strains. The chicks in both negative and positive control were not immunized with eBeam-killed Cp vaccine. The chicks in the negative control group were neither immunized nor challenged with Cp.

### 2.5. Statistical Analyses

Differences in the antibody response between vaccinated and unvaccinated groups were analyzed by one-way analysis of variance (ANOVA) (GraphPad Prism, San Diego, CA, USA). Differences in the amount of recovered Cp between vaccinated and unvaccinated groups were analyzed by one-way ANOVA (GraphPad Prism). A *p*-value less than 0.05 was statistically significant.

## 3. Results and Discussion

### 3.1. Characterization of E-Beam-Irradiated C. perfringens Cells 

**Figure 1:**
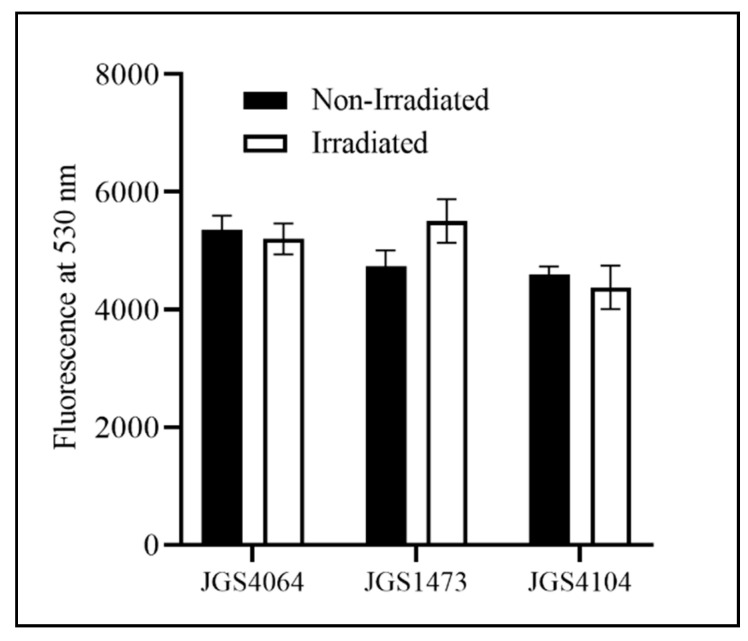
Fluorescence measured at 530 nm for eBeam-killed and non-irradiated (live cells) of three strains of *Clostridium perfringens* (JGS4064, JGS1473, and JGS4104) stained with *Bac*Light^™^ to determine the bacterial viability and cell wall integrity. Dark bar—non-irradiated CP cells; white bar—eBeam-killed CP cells (irradiated).

**Figure 2:**
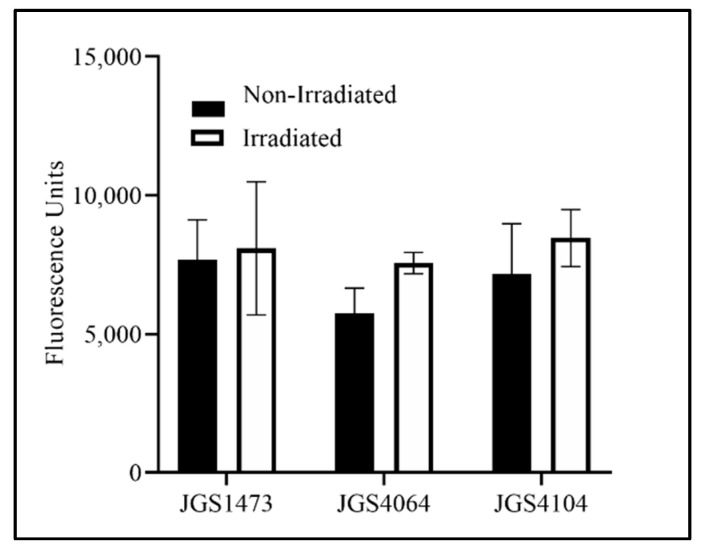
Fluorescence recorded for eBeam-killed and non-irradiated (live cells) of three strains of *Clostridium perfringens* (JGS4064, JGS1473, and JGS 4104) stained with alamarBlue^TM^ to determine the metabolic activity. Dark bar—non-irradiated CP cells; white bar—eBeam-killed CP cells (irradiated).

The D_10_ value of the individual strains was calculated to be between 0.83 kGy and 1.14 kGy (JGS1473: 1.14 kGy; JGS4104: 0.83 kGy; JGS4064: 0.98 kGy). Based on these values and because the typical titer of an overnight culture is approximately 10^8^ CFU/mL, a 10 kGy eBeam dose was chosen to inactivate Cp for the killed vaccine preparation. A similar fluorescence measurement in both LIVE/Dead^®^ BacLight™-stained eBeam-killed-Cp and untreated control Cp cells confirmed that the 10 kGy dose did not cause any damage to the cell membrane of eBeam-irradiated cells (Figure 1). The AlamarBlue assay was performed to check the viability and metabolic activity of the eBeam-killed-Cp cells. The results indicate that there were no differences in metabolic activity between eBeam-killed-Cp cells and (no eBeam treatment control) live Cp immediately after treatment (*p* > 0.05) (Figure 2).

**Figure 3:**
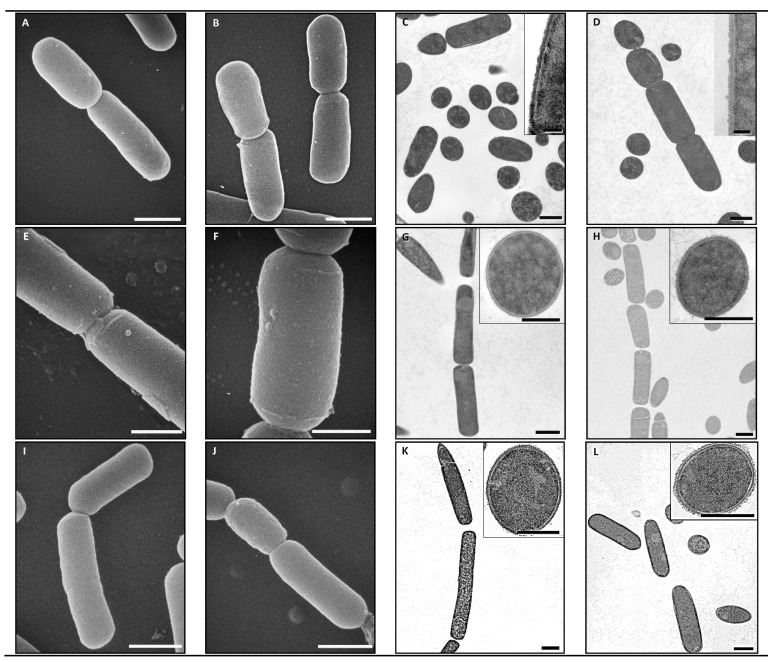
Scanning electron microscope (SEM) and transmission electron microscope (TEM) images of non-irradiated and eBeam-killed *C. perfringens* cells: (**A**) SEM of non-irradiated JSG4064 (bar = 1.0 µm); (**B**) SEM of eBeam-killed JSG4064 (bar = 1.0 µm); (**C**) TEM of non-irradiated JSG4064 (background bar = 1.0 µm; inset bar = 0.1 µm); (**D**) TEM of eBeam-killed JSG4064 (background bar = 1.0 µm; inset bar = 0.1 µm); (**E**) SEM of non-irradiated JSG1473 (bar = 0.5 µm); (**F**) SEM of eBeam-killed JSG1473 (bar = 0.5 µm); (**G**) TEM of non-irradiated JSG1473 (background bar = 1.0 µm; inset bar = 0.5 µm); (**H**) TEM of eBeam-killed JSG1473 (background bar = 1.0 µm; inset bar = 0.5 µm); (**I**) SEM of non-irradiated JSG4104 (bar = 1.0 µm); (**J**) SEM of eBeam-killed JSG4104 (bar = 1.0 µm); (**K**) TEM of non-irradiated JSG4104 (background bar = 1.0 µm; inset bar = 0.5 µm); (**L**) TEM of eBeam-killed JSG4104 (background bar = 1.0 µm; inset bar = 0.5 µm).

The ultrastructure examination of Cp strains under TEM and SEM revealed that there were no discernable differences in any of these isolates after eBeam exposure. The strains exhibited a range of morphologies that was consistent between the control and eBeam irradiated samples within a strain, but different between strains (Figure 3). Strain JGS4064 was observed as individual rods (Figure 3A–D), and strains JGS1473 and JGS4104 were observed as long chains of rods (Figure 3E–L). Bacterial spores were only observed in the control sample from isolate JGS4064 (Figure 3C). We have shown in this study that, when eBeam irradiation is performed at 10 kGy, there is no visible damage to the Cp cell membrane (Figure 3). This confirms that ionizing radiation mainly causes damages to nucleic acid [29,30]. The uniqueness of eBeam-based inactivation is that a vaccine can be developed where, even though the nucleic acid is shredded (preventive cell multiplication), the structural integrity of the bacterium ensures that there is little if any collateral damage to the cell membrane (Figure 3), thereby retaining the surface antigens/epitopes. Therefore, eBeam-killed vaccines have the safety of a killed vaccine yet retain the immunogenic potential of a live vaccine.

The eBeam-treated Cp cells did not multiply in either FTG broth or blood agar plates when incubated overnight anaerobically at 37 °C, or when incubated anaerobically at room temperature for up to 4 weeks. This suggested that the cells were lethally inactivated, preventing their multiplication even in favorable, nutrient-rich conditions. The enriched cecal contents from 2-week-old chicks that hatched from chicken SPF embryos inoculated with eBeam-irradiated Cp cells were also negative for Cp colonies. Both in vitro and in vivo attempts to resuscitate the eBeam-killed-Cp were unsuccessful, confirming that the 10 kGy eBeam dose used in this study killed the high titer (10^8^ CFU/mL) of bacteria in the vaccine preparation.

### 3.2. Protection against C. perfringens Colonization in Broiler Chickens

**Figure 4:**
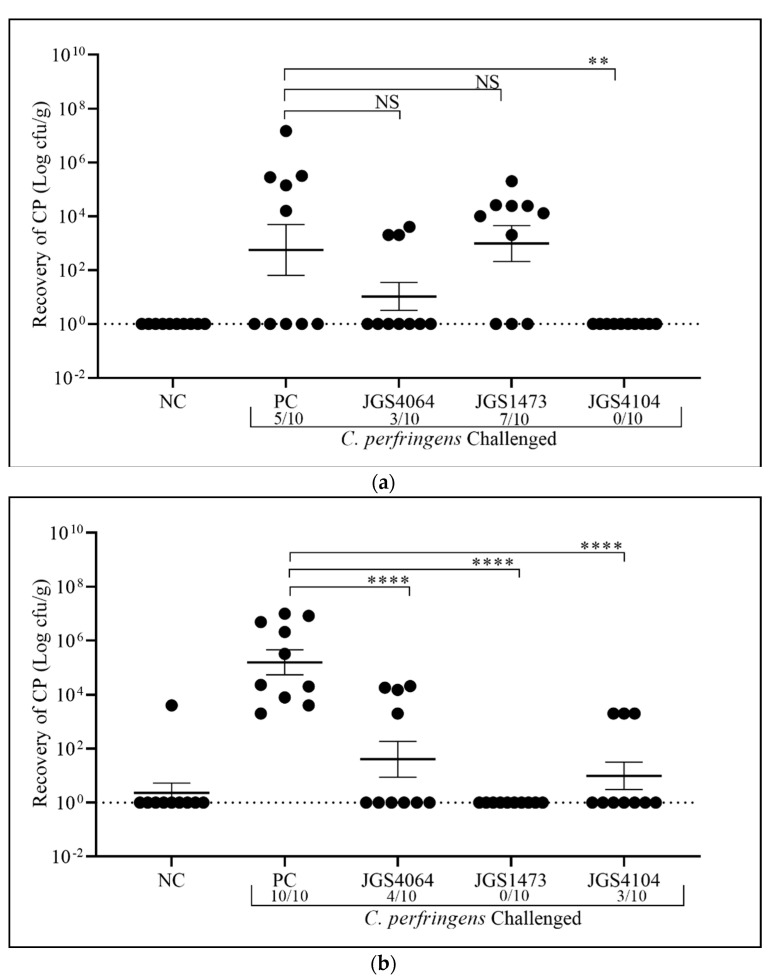
Prevention of *C. perfringens* (CP) colonization by eBeam-killed vaccine as determined by bacterial counts in ileal contents. Birds in all groups were inoculated with 1 × 10^7^ CFU of CP via oral gavage. Horizontal bar represents mean ± standard error. Birds were necropsied four days after post-challenge (day 22 for early-challenge and day 29 for late-challenge), and C. *perfringens* counts in ileal contents were determined (*n* = 10 in each groups). Significant difference in values, ** *p* < 0.01 and **** *p* < 0.0001, was determined by one-way analysis of variance (ANOVA) and NS = not significant. PC = Positive control; NC = Negative control. (**a**) Early challenge and (**b**) late challenge.

We did not observe any major clinical signs, morbidity, death, or weight loss in either the vaccinated or the unvaccinated birds (Table 2). It is widely known that obtaining gross lesion of NE consistently is a challenge under experimental conditions [37,38]. Moreover, the absence of clinical signs may be because of the absence of *Eimeria* in this study. Other studies have shown that co-infection of *Eimeria* species with Cp induced NE [39,40,41,42,43]. The ileal content of birds in the non-vaccinated and non-challenged negative control in the early-challenge were negative for Cp (Figure 4a), but one bird in the late-challenge was positive (Figure 4b). The colonization of Cp in negative control group of the late-challenge may be from the environment. The birds in the positive control (non-vaccinated, but challenged with Cp) had a higher burden of Cp in the late-challenge (mean of Cp 5.2 ± 0.5 log_10_ CFU/g) than the early-challenge (mean of Cp 2.8 ± 0.9); Figure 4a,b. Only 5/10 birds had Cp colonization in the early-challenge (Figure 4a), whereas all birds (10/10) were colonized with Cp in the late-challenge (Figure 4b). Though the birds in the positive control group were immunosuppressed via BursaVac and challenged with Cp for three consecutive days, only 50% of birds were positive for Cp in early-challenge, while 100% of the late-challenge group were positive for colonization. The reason for lower colonization in the early-challenge is unknown. 

Among birds immunized with eBeam-killed JGS4064, 30% (3 out of 10 birds) in the early-challenge (Figure 4a) and 40% (4 birds out of 10) in the late-challenge (Figure 4b) were positive for Cp colonization, indicating 70% and 60% protection, respectively. The mean Cp counts in the eBeam-killed-JGS4064 immunized birds in each challenge were lower than the positive control (early-challenge: 1.0 ± 0.5 and late-challenge: 1.6 ± 0.7 log_10_ CFU/g), but statistically not significant in the early-challenge (Figure 4a). The lack of statistical significance in the early challenge group when compared with the positive control group is likely due to a low number of Cp-positive birds in the positive control group. The late-challenge had a significantly (1.6 ± 0.7 log_10_ CFU/g, *p* < 0.006) reduced number of Cp in the positive birds (Figure 4b).

Among birds immunized with eBeam-killed-JGS1473, 70% (7 out of 10 birds) in the early-challenge (Figure 4a) and 0% (0 birds out of 10 birds) in the late-challenge (Figure 4b) were positive for Cp colonization, indicating 30% and 100% protection, respectively. The mean Cp counts in the eBeam-killed-JGS1473 immunized birds of early-challenge were higher (2.98 ± 0.67 log_10_ CFU/g) than the positive control, but not statistically significant (Figure 4a), whereas Cp colonization in the late-challenge was significantly (*p* < 0.0001) reduced (0 log_10_ CFU/g) (Figure 4b).

Among birds immunized with eBeam-killed-JGS4104, 0% (0 out of 10 birds) in the early-challenge (Figure 4a) and 30% (3 out of 10 birds) in the late-challenge (Figure 4b) were positive for Cp colonization, indicating 100% and 70% protection, respectively. The mean Cp counts in the eBeam-killed-JGS4104 immunized birds in each challenge were lower than the positive control in both challenges and statistically significant (Figure 4a,b) in the early-challenge (0 log_10_ CFU/g, *p* < 0.004) and late-challenge (0.99 ± 0.5 log_10_ CFU/g, *p* < 0.001), respectively. The reason for the presence of three Cp positive birds in the late-challenge could be the colonization of non-vaccine strains from the environment.

All three strains used in the study provided protection when challenged with the homologous strains, except Cp JGS1473. In this strain, birds were less protected in the early challenge, but showed 100% protection in the late challenge. This may indicate that the immune response of this strain is delayed when compared with the other strains used in this study. Interestingly, JGS1473 was initially isolated from the microflora of a healthy bird, while JGS4104 and JGS40164 were isolated from a turkey and chicken with NE, respectively [32].

**Figure 5:**
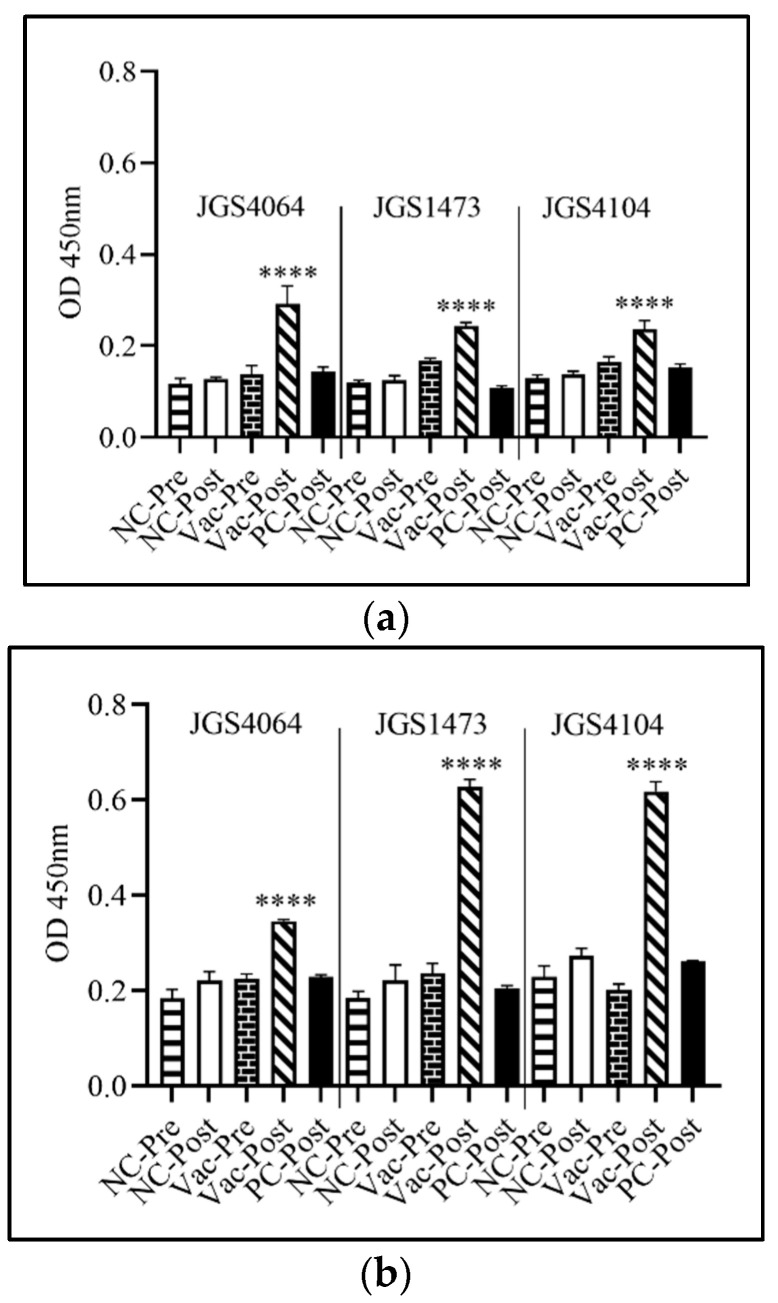
IgY response to CP strains: JSG4064, JSG1473, and JSG4104 using enzyme-linked immunosorbent assay (ELISA). Day 18 chicken embryos were immunized in ovo with either eBeam-killed-JSG4064, eBeam-killed-JSG1473, or eBeam-killed-JSG4104. Pre-challenge sera were collected on day 18 for early-challenge and day 25 for late-challenge. Post-challenge sera were collected on day 22 for early-challenge and day 29 for late-challenge. Each bar represents mean ± SEM. Asterisks indicate a statistically significant difference relative to the post-challenge positive control birds (**** *p* < 0.0001) determined by one-way ANOVA. OD = optical density (**a**) Early challenge and (**b**) late challenge.

The serum IgY level was significantly (*p* < 0.0001) lower in both the negative control (unvaccinated and unchallenged) and positive control (unvaccinated, but challenged) birds when compared with the birds immunized with individual eBeam-killed vaccine (Figure 5a,b). In general, higher IgY levels were observed in the post-challenged birds in all three immunized groups than in the negative and positive control. IgY levels were significantly higher after challenge in all groups. Furthermore, IgY levels in birds in the late-challenge group had higher IgY than in the early-challenge group (Figure 5a,b). The increased protection against Cp colonization (Figure 4b) in all three immunized groups and in later challenged groups could be due to the presence of elevated levels of IgY (Figure 5b).

We had previously developed an eBeam-killed vaccine against *S*. Enteritidis [29]. We obtained similar results of reduced colonization of *S*. Enteritidis in the vaccinated birds. We had also observed that the eBeam-killed vaccine is antigenic and induces innate immunity [44]. Other bacterial vaccines (*Brucella melitensis, Listeria monocytogenes*, *Rodentibacter pneumotropicus*, *Staphylococcus aureus*, and *Streptococcus pneumoniae*) developed using ionizing radiation showed higher humoral immune responses and protection against bacterial infection in humans and animals [31,45,46,47,48,49,50,51]. Whole cells inactivated vaccines have been shown to provide cross protective immunity against other strains [52,53]. Though the Cp challenge in this study did not produce clinical signs for NE, the eBeam-killed vaccine reduced the colonization of Cp compared with the non-vaccinated control, suggesting that eBeam technology could be a good tool to kill/inactivate the bacterial pathogen without damaging the epitopes. As each Cp strain produces a variety of toxins, vaccinating birds with an individual strain might not protect against other strains. Moreover, Cp strains can also transfer their plasmids by horizontal gene transfer, thereby converting non-pathogenic strains to pathogenic strains [54]. It is critically important to perform further studies to understand the mechanism of vaccine protection, test cross protection abilities, as well as use a mixture of virulent strains in the vaccine preparation. As Cp is not only a poultry pathogen, but also considered as a foodborne pathogen that causes foodborne illnesses in the United States and other countries [55,56], controlling Cp in broiler chickens by vaccination will not only reduce the economic loss, but also reduce human foodborne illnesses.

The benefits of developing vaccines using eBeam technology are that it is chemical-free, highly scalable, and can be very precise in delivering the eBeam doses as well as assure complete inactivation of large volume lots. Presently, several irradiated viral and bacterial vaccines have been developed that have been shown to be more efficacious than other vaccines prepared by other technologies [57,58,59,60,61,62]. Electron beam technology platforms are continually improving in terms of robustness, modularity, and versatility in terms of beam power and electron energies [63]. Another major advantage of eBeam-killed vaccines is that their potency can be maintained for extended periods of time. We have recently reported that, even after 30 days at room temperature, the immunoreactivity profile of eBeam-inactivated *Salmonella* Typhimurium was very similar to that of live, unexposed *Salmonella* Typhimurium [64]. The cost of implementing this technology for the veterinary industry will depend on the operating costs and the amortized capital costs [63]. However, low-energy eBeam dosing systems are already in development and have been demonstrated to be suitable for developing viral vaccines [52]. Once low-energy eBeam and similar technologies are commercially available, the costs of this technology per unit vaccine dose would become clearer.

## 4. Conclusions

Vaccination is the preferred alternative to control Cp colonization in broiler chickens than antibiotics mixed infeed/water. Vaccines have been used to successfully control clostridial diseases in livestock animals [62]. In this study, we have developed killed Cp vaccines using eBeam technology against three individual Cp strains. The vaccines were delivered in ovo to day 18 embryos and good protection against colonization of Cp in early and late challenge was observed, except for one strain (JGS1473), which had lower protection in the early challenge. Though we did not find significant clinical signs for NE, the vaccine significantly reduced the colonization of Cp (Figure 5a,b). The advantage of using an eBeam-killed vaccine is that it reduces the potential risk of pathogen survival within the poultry houses and posing a threat to both humans and subsequently a lot of birds. Overall, the above-described approach to prevent Cp colonization in chickens can be potentially paradigm-shifting in terms of ability to quick develop vaccines against prevailing strains and the ability to rapidly scale the inactivation step.

## Figures and Tables

**Table 1:** Experimental design. FTG, fluid thioglycolate broth.

Group	Treatment Groups	In ovo	Challenge	Number of Birds	
				Early-Challenge	Late-Challenge
1	Neg Control	FTG medium	No challenge	25	25
2	Positive Control	FTG medium	JGS4064, JGS4104, JGS1473	25	25
3	Challenge-Eyes	FTG medium	Bursa-Vac	25	25
4	eBeam killed JGS4064	vaccine	JGS4064	25	25
5	E-Beam killed JGS4104	vaccine	JGS4104	25	25
6	E-Beam killed JGS1473	vaccine	JGS1473	25	25

**Table 2:** (**a**) Response of birds to early-challenge with various *C. perfringens* (CP) strains. (**b**) Response of birds to late-challenge with various *C. perfringens* (CP) strains.

(a)			
Treatment	Birds with Gross Lesions/Total Birds (%)	Average Lesion Score	Average Weight of Birds before Necropsy (kg)
JGS1473	1/25 (4)	0.08	0.76
JGS 4064	2/25 (8)	0.08	0.75
JGS 4104	3/25 (12)	0.12	0.79
Positive control	11/25 (44)	0.6	0.81
Negative control	0/25 (0)	0	0.84
**(b)**			
**Treatment**	**Birds with Gross Lesions/Total Birds (%)**	**Average Lesion Score**	**Average Weight of Birds before Necropsy (kg)**
JGS1473	4/25 (16)	0.16	1.22
JGS 4064	4/25 (16)	0.16	1.23
JGS 4104	3/25 (12)	0.12	1.26
Positive control	6/25 (24)	0.28	1.24
Negative control	1/25 (4)	0.04	1.23

## Data Availability

This study did not report any data.
